# P-685. Investigation of Influenza antibody transfer following maternal vaccination using the PREVAIL Birth Cohort

**DOI:** 10.1093/ofid/ofae631.881

**Published:** 2025-01-29

**Authors:** Allison R Cline, Shannon C Conrey, Ardythe L Morrow, Mary A Staat, Danielle Fayad, Samantha M Olson, Daniel C Payne, Angela P Campbell

**Affiliations:** University of Cincinnati College of Medicine, Cincinnati, Ohio; University of Cincinnati College of Medicine, Cincinnati, Ohio; University of Cincinnati College of Medicine, Cincinnati, Ohio; Cincinnati Children’s Hospital Medical Center, Cincinnati, Ohio; Cincinnati Children's Hospital Medical Center, Cincinnati, Ohio; Centers for Disease Control and Prevention, Atlanta, Georgia; CDC, Decatur, Georgia; Centers for Disease Control and Prevention, Atlanta, Georgia

## Abstract

**Background:**

Seasonal influenza vaccine is recommended during pregnancy; however, data are limited on transplacental antibody transfer. We used the PREVAIL Cohort of healthy, term infants and their mothers in Cincinnati, Ohio, to describe hemagglutination-inhibition (HAI) titers in pregnancy and measure the association between maternal and cord HAI relative to maternal vaccination during pregnancy.

**Table 1.**
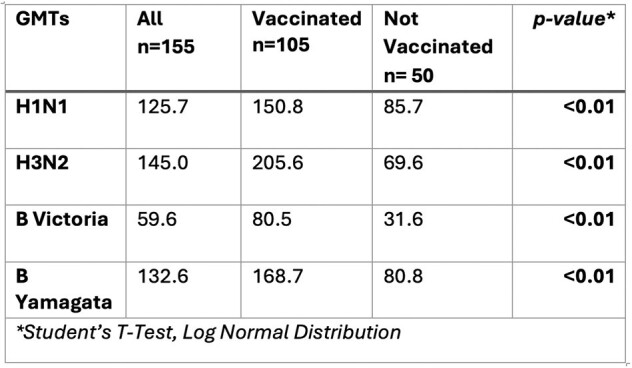
Maternal GMT by Vaccination Status and by Influenza A Subtypes and Influenza B Lineages

**Methods:**

Maternal immunization status was abstracted from medical charts/state registries. HAI titers were measured in maternal blood collected in the third trimester, and in cord blood. HAI > 1:40 were considered seroprotective. Paired T tests (log normal distribution) and Fisher’s exact tests were used to compare geometric mean titers (GMT) and proportion of participants with seroprotective HAI, respectively, for each influenza subtype/lineage by vaccination status. Association between maternal and cord HAI was assessed using Spearman correlation and Maternal HAI/Cord HAI ratios.

**Table 2.**
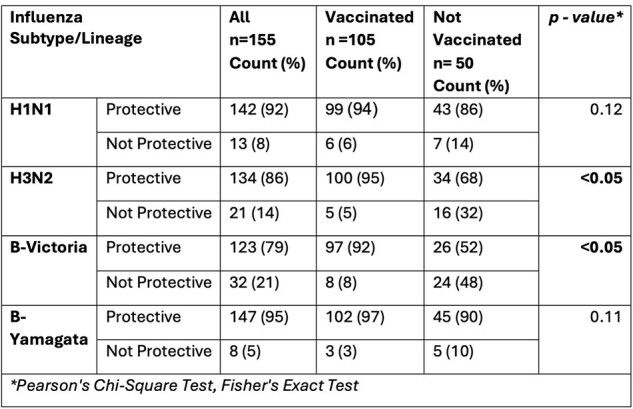
Maternal Seroprotection by Vaccination Status and by Influenza A Subtypes and Influenza B Lineages

**Results:**

155 mothers were included and delivered in the 2017-2018 influenza season (July 1^st^ 2017-June 30^st^ 2018); H3N2, H1N1 and B viruses circulated this season. 105 mothers received influenza vaccine during or 4 months prior to pregnancy. Vaccinated mothers had significantly higher GMTs for all influenza subtype/lineage compared to unvaccinated mothers (p< 0.005) (Table 1). A significantly higher proportion of vaccinated mothers exhibited seroprotective titers against influenza subtype/lineage H3N2 and B-Victoria compared to unvaccinated mothers (p< 0.05) (Table 2). There was a significant correlation between maternal and cord blood HAI for all subtype/lineage (r > 0.85, p< 0.05) (Figure 1). Cord GMTs tended to exceed maternal GMTs, with statistically significant differences in Influenza B lineage (p< 0.05) (Table 3). More than 75% of the mother-infant pairs exhibited transplacental antibody transfer ratios ≤ 1.

**Table 3.**
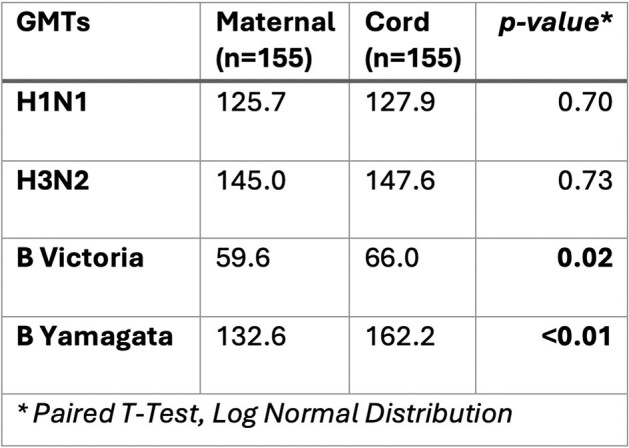
Maternal GMT and Cord GMT by Influenza A Subtypes and Influenza B Lineages

**Conclusion:**

Vaccinated mothers displayed protective influenza antibodies levels which were significantly higher than those found in unvaccinated mothers. Maternal HAI was strongly correlated with cord HAI, suggesting efficient transplacental transfer. Moving forward, investigation of factors influencing this transfer will be undertaken.

**Figure 1.**
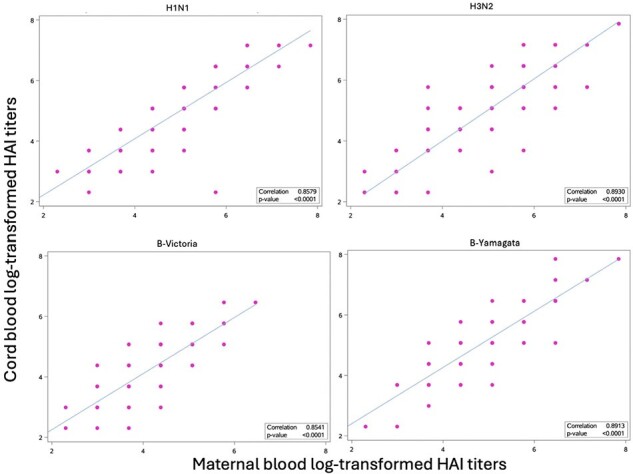
Scatter plots of maternal vs cord log-transformed HAI titers

**Disclosures:**

**Mary A. Staat, MD, MPH**, Cepheid: Grant/Research Support|Merck: Grant/Research Support|Pfizer: Grant/Research Support|Up-To-Date: Honoraria

